# Rethinking areas of endemism and barriers: perspectives for a causal historical biogeography and a critique of Schultz and Cracraft (2024)

**DOI:** 10.1111/cla.70030

**Published:** 2026-03-06

**Authors:** Augusto Ferrari

**Affiliations:** ^1^ Laboratório de Entomologia, Sistemática e Biogeografia—LESB, Instituto de Ciências Biológicas Universidade Federal do Rio Grande—FURG Rio Grande Brazil; ^2^ Programa de Pós‐Graduação em Biologia Animal—PPG‐BAN Universidade Federal do Rio Grande Sul, UFRGS Porto Alegre Brazil

## Abstract

Historical biogeography faces a persistent conceptual and methodological dilemma concerning the nature of its central analytical units. Using the recent proposal by Schultz and Cracraft (*Cladistics* 40, 653) as a catalyst, this article critiques the argument that causal inference necessitates the replacement of areas of endemism with barriers, contending that such a forced dichotomy is both artificial and reductionist. It is postulated that modern methodological advances allow areas of endemism to be rigorously treated as dynamic and quantitatively testable hypotheses, thereby overcoming long‐standing objections that classify them as mere descriptive constructs. Conversely, an exclusive focus on barriers fundamentally underestimates the multifactorial nature of diversification, neglecting the structural role of dispersal and the complex spatiotemporal dynamism of isolating forces. Effective inference in historical biogeography demands an explicitly pluralistic and process‐agnostic perspective, recognizing areas and barriers as complementary, intersecting elements. This integrative approach is essential for accurately capturing the emergent complexity of biogeographic patterns, moving the discipline beyond the limitations of unifocal causal models.

## Introduction

A long‐standing conceptual and methodological dilemma in historical biogeography concerns the nature and explanatory scope of its traditional analytical units (Henderson, 1991; Hovenkamp, 1997; Linder, 2001; Arias et al., 2011; Ferrari, 2017; Schultz and Cracraft, 2024). Historically, the field developed by describing spatial patterns, especially through the identification of areas of endemism, which are defined by the congruent and non‐random restricted distributions of taxa (Morrone, 1994). Such approaches have contributed greatly to understanding historical relationships among biotas, but their ontological status and explanatory power have increasingly been questioned (Anderson, 1994; Hovenkamp, 1997). Schultz and Cracraft (2024) propose to tackle this dilemma with a mechanistic historical biogeography perspective, advocating direct investigation of the causal mechanisms behind organisms' distribution histories. In their view, areas of endemism are considered limited conceptual constructs that should be replaced by geographic barriers. These barriers, conceived as concrete entities bounded in space and time, would allow more rigorous hypothesis testing and a clearer grasp of the evolutionary processes involved.

Schultz and Cracraft (2024) emphasize that the use of analytical units in historical biogeography arose mainly through the adoption of areas of endemism, often treated as ‘characters optimized’ on phylogenies. In the section ‘Areas of endemism confound biogeographic history’, the authors argue that areas of endemism face three fundamental problems: they are not discrete historical entities, they are largely human constructs shaped by methodological choices, and they are inadequate for analyses aiming at causal explanations. From their perspective, approaches that treat areas as predefined characters or optimization units yield results that are essentially descriptive, thereby restricting the explanatory scope of historical biogeography. According to these authors, understanding biogeographic history requires moving beyond the mere cataloguing of spatial patterns, urging a renewed focus on the relationship between geography and the origin of species. Assuming that allopatric speciation is the predominant mode of diversification, they argue that we must investigate the origin of that allopatry by linking it to concrete events in Earth history. Thus, geographic barriers would become the central units of analysis because they allow diversification events to be tied directly to specific causal processes, providing a more solid, mechanism‐based foundation for biogeographic hypotheses.

While Schultz and Cracraft (2024) offer a necessary reassessment of the field's foundations, they also provide an opportunity to move toward a more integrative synthesis. My argument builds on the view that portrayals of areas of endemism have often overlooked recent methodological advances and relied on a simplified description of area‐based approaches. Rather than seeing areas and barriers as mutually exclusive or as substitutes for one another, I suggest that both face similar conceptual and operational challenges and can be more productively understood as complementary perspectives. A constructive path forward is to recognize the plural, dynamic, and emergent nature of historical biogeography, integrating different analytical units while remaining mindful of their explanatory limits.

## Areas of endemism reappraised: ontology, methods, evidence and homology

Schultz and Cracraft (2024) conflate ontological, methodological and explanatory questions about areas of endemism. Conceptually distinct concerns, including fuzzy boundaries, temporal instability and limited possibilities for causal inference, are treated as a single problem, while recent advances in criteria for area recognition, procedures for assessing area support, and the integration of temporal, environmental and phylogenetic evidence receive little attention. This lack of clarity whether their target is the ontological status of areas, the methodological standards for their delimitation or the inferential links provided by area optimization ultimately weakens the force of their critique.

### Ontology

The ontological argument questioning the validity of areas of endemism rests on significant conceptual challenges. Yet, the difficulty of diagnosing areas of endemism or inconsistencies in their delimitation, presence of diffuse boundaries or the dependence on time and scale do not constitute evidence against their existence as historical entities, just as the complexity of identifying cryptic species does not invalidate their biological reality (see Velasco, 2008). This line of criticism conflates ontological questions, which address the actual existence of such areas, with operational questions, which concern the methods used to recognize them. Areas of endemism are not ontologically given entities but emergent patterns resulting from the superimposition of species' distributions shaped by historical and ecological processes (Cracraft, 1985; Harold and Mooi, 1994; Morrone, 2009).

From an ontological perspective, Parenti and Ebach (2009) argue that areas of endemism are not arbitrary constructs but rather the natural units of comparative biogeography, defined by the shared historical distributions of their constituent taxa. Central to this view is the concept of area homology, which parallels organismal homology by treating areas as entities that can be compared through their common biotic history. Importantly, these areas are inherently dynamic, with boundaries that shift in response to climatic, geological or ecological changes, yet this variability does not undermine their ontological status. Thus, far from being arbitrary constructions, biotic areas reflect the real distribution of organisms, which may or may not have recognizable physical boundaries but are always delimited by the taxa that inhabit them and not others. The comparison of homologous areas enables the inference of biogeographic patterns and the evaluation of their congruence with geological history, reinforcing their status as testable historical hypotheses. Biogeography can be approached from two complementary conceptual perspectives: an evolutionary perspective, which focuses on modelling the distributional histories of taxa and proposing underlying mechanisms such as competition, predation, dispersal and vicariance; and a systematic perspective, which seeks to identify area homologies and to classify these homologous areas based on shared biotic relationships (Hovenkamp, 1997; Ebach and Morrone, 2005; Fattorini, 2008).

Much of Schultz and Cracraft's (2024) critique builds on the view of Crother and Murray (2011, 2013), who treated areas of endemism as ‘individuals’ defined by unique combinations of species. From that premise, Schultz and Cracraft conclude that areas of endemism are not discrete historical entities, but are largely human constructs with little explanatory power; however, this argument revives points that have already been challenged and ignores key counterarguments by Hovenkamp (2013). Hovenkamp (2013) demonstrated that Crother and Murray's approach suffers from serious operational and conceptual shortcomings, including difficulties in interpreting PAE results, the production of an artificial nested hierarchy of areas, and the absence of any theoretical justification for treating such nestedness as natural, and their criteria are generally regarded as impractical to apply.

### Methodological advances in the recognition of areas of endemism

Much has changed since the earliest quantitative methods for identifying areas of endemism, whether in the criteria used to delimit areas, or in the evaluation of results, or even in the quality of the data employed in the analyses. The definition of an area of endemism based on a single species, or even on two or more species, constitutes weak evidence for its individuation (Schultz and Cracraft, 2024). The concept of area of endemism is fundamentally based on the notion of restricted distributional congruence among at least two taxa, since fewer than two would not yield a discernible pattern (Platnick, 1991; Morrone, 1994; Fattorini, 2017; but see Parenti and Ebach, 2009). As with phylogenetic analyses, where branches may show varying levels of support and researchers often set thresholds (e.g. Hillis and Bull, 1993), such as preferring 90% bootstrap or jackknife support over 70%, biogeographers retain similar flexibility in defining the amount of evidence for recognizing an area of endemism. The number of species supporting an area, as well as the degree to which their distributions conform to it, reflects directly the strength of the hypothesis rather than its validity. Importantly, some authors do not restrict the recognition of areas of endemism to minimal evidence, but instead treat the level of support as a gradient that informs confidence, not existence. For instance, Hoffmeister and Ferrari (2016) required at least six species to define an area of endemism, whereas other authors accept as few as two taxa with congruent restricted distributions (Crother and Murray, 2011).

Analytical approaches, such as Endemicity Analysis (Szumik et al., 2002; Szumik and Goloboff, 2004), implemented in NDM/VNDM (Goloboff, 2004), allow the adjustment of parameters that regulate the criteria of distributional congruence among species. Weighting factors (e.g., Fi and Fa) modulate the influence of inferred or assumed presences, while others (Fo, Fd, Fn) penalize external records according to their distance from the candidate area, thereby reducing artificial fits of widespread or irregularly distributed species. Additional adjustments, such as minimum species scores, define thresholds for the minimum degree of congruence required, ensuring that only taxa with strong distributional fit contribute significantly to the endemicity index (Szumik and Goloboff, 2004). Subsequent developments include reanalysis and resampling to assess the stability of delimited areas, analogous to phylogenetic support (Casagranda and Goloboff, 2019). In combination, these features make Endemicity Analysis both tunable and testable, parameters can be calibrated, support quantified, and assumptions made explicit, thereby countering claims of fragility or arbitrariness and framing areas of endemism as hypotheses open to quantitative corroboration rather than fixed constructs.

Research on identifying areas of endemism has advanced by examining how method‐specific parameters shape results, and by comparing different approaches on the same datasets to assess congruence among solutions. This has enabled the separation of robust, recurrent patterns from procedure‐dependent ones, improving the reliability of proposed areas of endemism, the detection of method‐specific artefacts, and the delineation of clearer bounds for biogeographic inference. Numerous studies explore the effects of grid‐cell size or the influence of categories of the geographic interpolation of endemism (e.g. Escalante and Morrone, 2002; Szumik et al., 2012; Sandoval and Ferro, 2014; Garraffoni et al., 2017; Lago‐Barcia et al., 2020), evaluate data‐extrapolation procedures (e.g. Hoffmeister and Ferrari, 2016; Ferrari et al., 2022), and incorporate presence records derived from species distribution models to mitigate sampling bias and more adequately represent taxon distributions (e.g. Rovito et al., 2004; Escalante et al., 2013; Prieto‐Torres et al., 2019). In NDM/VNDM, comparative sensitivity analyses explore multiple parameter configurations and employ a metaconsensus to integrate results; only areas consistently recovered are retained, indicating robustness to parameter choice and, consequently, greater inferential reliability (Hoffmeister and Ferrari, 2016; Alvares et al., 2022; Ferrari et al., 2022).

### Chorotype, area of endemism and homology

Regardless of the method used to identify areas of endemism or detect shared spatial patterns, all approaches rely on establishing a criterion of distributional congruence (e.g. Morrone, 1994; Hausdorf and Hennig, 2003; Szumik and Goloboff, 2004; Oliveira et al., 2015; Edler et al., 2017). Analytical parameters can be adjusted to penalize incongruences to different degrees, producing candidate spatial units. While methods such as Parsimony Analysis of Endemicity (PAE, Morrone, 1994) or NDM/VNDM are designed to recognize areas of endemism in the historical sense, their results may also represent chorotypes (e.g. Alvares et al., 2022), sets of taxa with similar distributions, without implying direct causal links between pattern and origin (Fattorini, 2016). This distinction between areas of endemism (historically meaningful entities) and chorotypes (distributional clusters without causal assumptions) is central to maintaining coherence between the ontological status of a unit and the operations used to detect it. Some methods deliberately avoid the term ‘areas of endemism’, preferring alternatives such as ‘biotic elements’ (Hausdorf and Hennig, 2003) or ‘bioregions’ in the Infomap framework (Edler et al., 2017). This choice reflects their focus on distributional patterns (i.e. to summarize recurrent species range) rather than causal–historical interpretation, and the fact that they do not account for species restrictedness, a key criterion for the formal recognition of areas of endemism.

As Schultz and Cracraft (2024, p. 665) note, the idea that endemism can be defined solely by a ‘unique combination of species’ is problematic, since the taxa within a region may not share a common history, even though history underlies most conceptualizations of areas of endemism. This highlights the need to separate the ontological and causal status of areas from the operational procedures used to identify them. Initial recognition is largely phenomenological (i.e. primary biogeographic homology), while corroboration with phylogenetic evidence provides secondary, process‐based hypotheses (Morrone, 2001; Fattorini, 2017). Thus, distinguishing between areas of endemism, chorotypes and primary homologies is methodologically necessary, as conflating them risks oversimplifying analyses and misdirecting criticisms (see Fattorini, 2016, 2017; Morrone, 2020). Importantly, the same caveat applies to other biogeographic contexts, for example, the co‐occurrence of pairs of sister taxa along a barrier does not prove that they were shaped by the same historical event (see Fattorini, 2008). The central challenge lies in establishing spatial and temporal congruence, an issue inherent to both areas and barriers (see above).

### Areas of endemism and time

The concern that areas of endemism are difficult to define because their boundaries and durations vary is legitimate (Schultz and Cracraft, 2024, p. 655), yet shifting the focus to barriers does not resolve this issue, since barriers themselves are dynamic and often lack precise limits. In practice, available methods for recognizing areas of endemism correspond to recognizing a horobiota, a temporal snapshot of a biota, through an inferential sequence that begins with describing distributional patterns, proceeds by testing temporal congruence (e.g. with dated phylogenies), and culminates in hypotheses about historical causes (Morrone, 2001, 2009, 2020). Within this framework, areas of endemism function as preliminary hypotheses of biotic assembly that may be further refined into cenocrons, subsets of taxa sharing a common biogeographic history and constituting testable historical entities. As emphasized by Noguera‐Urbano (2016), however, these units should not be understood as purely spatial constructs, but as spatiotemporal entities shaped by synchronous and asynchronous divergence events, reflecting the threefold interplay of space, time, and form in biogeographic history. Areas of endemism gain ontological robustness when their biological distinctiveness aligns with shared geological histories, forming natural biogeographical units rather than arbitrary constructs (Michaux, 2010).

Recent studies have strengthened this perspective by explicitly incorporating the temporal dimension into areas of endemism. Bitencourt and Rapini (2013) showed that spatial patterns in the Espinhaço Range, when combined with phylogenetic evidence, can reveal both historical continuity and shifts through time. Gámez et al. (2014) demonstrated that many areas of endemism persist despite climatic and environmental change, underlining their historical resilience. Pinilla‐Buitrago et al. (2018) advanced this by integrating dated phylogenies and temporal slices into endemicity analysis, revealing how endemism can emerge at different temporal scales and how spatial–temporal congruence reinforces inference. More recently, Caujapé‐Castells et al. (2022) incorporated time explicitly into endemicity analysis in the Canary Islands, using calibrated phylogenies to link divergence events to geological phases of island formation. Their approach illustrates how areas of endemism can be understood as hypotheses grounded in both spatial patterns and evolutionary timing, providing a framework to evaluate not only where but also when endemic assemblages originated and persisted. Collectively, these works illustrate how areas of endemism, far from being arbitrary constructs, embody the dynamic yet historically grounded nature of biotic assembly, providing robust and testable hypotheses. As Nihei's (2008) notion of ‘dynamic endemism’ makes clear, shifting boundaries reflect the dynamism of Earth and life, not the unreality of these areas.

Finally, Szumik et al. (2019) demonstrated that neighbouring areas of endemism frequently overlap and are rarely characterized by sister taxa, directly challenging the long‐standing assumption that vicariant areas should display reciprocal monophyly. This finding undermines the expectation that ‘sister areas’ must be supported by sister taxa and highlights the lack of phylogenetic evidence validating that assumption. The authors further emphasize that vicariance is not the sole process responsible for shaping areas of endemism and reiterate Hovenkamp's (1997) broader point: the persistent analogy between taxa and areas has fostered conceptual and methodological misunderstandings in historical biogeography.

### Areas as biogeographic units

In historical biogeography, inferences from area‐based methods are contingent on how geographic space is encoded—specifically, on the partitioning of species distributions into operational analytical units. Event‐based methods (e.g. Ancestral Areas, DIVA, TreeFitter) and parametric approaches in historical biogeography (e.g. BayArea, DIVALIKE, DEC and the +J parameter variants implemented in BioGeoBEARS) require some level of spatial discretization of taxon distributions (see Ferrari, 2017 and references therein). For some authors, a stepwise procedure is unnecessary in parametric biogeography, since the choice of analytical units should be dictated by the research question itself rather than by predefined criteria; accordingly, these methods do not presuppose areas of endemism, but can operate with any type of discrete spatial unit (Ree and Sanmartín, 2009; Matzke, 2025). A substantial part of the literature explicitly acknowledges alternative units, such as biotic elements, chorotypes, regionalization schemes, geological areas, or bioregions, as valid analytical entities (Ferrari, 2017).

It is not entirely clear whether Schultz and Cracraft critique is aimed specifically at those ‘area‐optimization’ approaches or at the use of areas of endemism as primary units in general. Describing model‐based approaches as ‘optimizing areas as characters’ (Schultz and Cracraft, 2024, p. 656) is an oversimplification. Event‐based parsimony methods, such as DIVA or TreeFitter, indeed rely on minimizing or maximizing the total cost of biogeographic events (vicariance, duplication, dispersal, extinction) (Sanmartín, 2010, 2021). In contrast, parametric probabilistic methods aim to estimate ancestral ranges rather than optimize fixed “areas,” using continuous‐time Markov chain models that describe range dynamics over phylogenetic time, explicitly incorporating processes such as dispersal, extinction, vicariance, and founder events (Ronquist and Sanmartín, 2011; Matzke, 2013a, 2013b, 2014). These models estimate parameters from the data rather than relying on *ad hoc* cost assignments, and operate in a framework that is conceptually distant from treating areas as simple characters. A truly ‘character‐based’ approach would resemble ancestral area reconstruction via Fitch optimization, which has been shown to violate key biogeographic assumptions and is generally discouraged in modern analyses (Ronquist and Sanmartín, 2011; Sanmartín, 2021). Thus, the limitations sometimes attributed to ‘area‐optimization’ approaches do not stem inherently from the use of areas of endemism, nor are areas of endemism restricted to serving as predefined analytical units in historical biogeography.

An important clarification is that many historical biogeographic studies that “use areas” actually rely on regionalization schemes, such as Morrone's areas (Morrone, 2014; Morrone et al., 2022) or global ecoregions (Dinerstein et al., 2017). Although these units may, to some extent, reflect patterns of endemism, they are not typically delimited using methods that primarily operationalize endemism. Biogeographical regionalization denotes a hierarchical organization of geographic areas based on their biological composition. These units are characterized by unique assemblages of endemic taxa and communities, reflecting the distribution of life moulded by interacting physical and biological forces through time (Morrone, 2018). In fact, few studies of ‘area optimization’ employ areas of endemism delineated by explicit methods, because a common outcome of endemism‐identification procedures is the recovery of overlapping areas. This overlap complicates the selection of analytical units and the use of those areas as terminals in area‐cladogram analyses. Areas of endemism can serve as a foundation, or a refinement, for regionalization schemes, and conversely, such schemes can guide the search for areas of endemism (Morrone, 2018). Nevertheless, some regionalization approaches (e.g. similarity/dissimilarity analyses using indices such as Simpson's, coupled with agglomerative clustering algorithms like UPGMA) do not operationalize endemism explicitly (Machado et al., 2024). Critiques of ‘area‐based’ biogeography must specify whether their target is (i) the concept and detection of areas of endemism, (ii) the construction of regionalization schemes, or (iii) the treatment of area units as characters in historical analyses.

## The limits of a barrier‐driven approach to mechanistic biogeography

Identifying the causes of diversification requires addressing the origins of barriers that create allopatry through processes of Earth history (Schultz and Cracraft, 2024). While barriers are important in shaping allopatric speciation, this perspective oversimplifies the phenomenon, which can also result from range dynamics, ecological shifts, and stochastic events. Equating allopatry exclusively with barrier formation imposes a reductionist framework that neglects the multifactorial nature of historical biogeography, since barriers are not limited to physical structures but may also be climatic, ecological, or functional (Smith et al., 2014; Zullini, 2018). As Hausdorf (2025) argues in the context of speciation, effective discontinuities do not arise from discrete units, whether genes, genomes, or physical barriers, but emerge from the contingent coupling of multiple genetic, ecological, and developmental factors. Just as no single mechanism governs the formation of species boundaries, no single geographical process is sufficient to account for the spatial structuring of taxa. Biogeographical patterns are emergent outcomes of heterogeneous processes acting across scales, and attempts to impose a unilinear causal axis obscure precisely the complexity that requires explanation.

Allopatric speciation is widely recognized as the predominant mode of diversification across the Tree of Life, yet it should be regarded primarily as a pattern of distributional discontinuity rather than a mechanism per se (Wiens, 2024). Allopatry may arise through classical vicariant events, in which emerging barriers subdivide populations, or through dispersal processes, either across pre‐existing barriers or toward isolated regions where no direct physical division was initially present (Fattorini, 2008; Morrone, 2020). The isolating forces underlying these scenarios extend beyond purely geographical features: ecological and functional barriers, such as climatic gradients, habitat discontinuities, host specialization in insects or reproductive timing in marine organisms, can equally restrict gene flow and drive divergence (Forbes et al., 2017; Zullini, 2018; Wiens, 2024). Empirical evidence illustrates this diversity of pathways: insects frequently speciate via host‐driven isolation; amphibians and reptiles along ecological or climatic gradients; freshwater fishes through riverine connectivity and physiological tolerance limits; and many marine taxa through ecological or behavioural mechanisms even in the absence of long‐term physical barriers. Importantly, the effectiveness of any barrier is not absolute, but contingent on intrinsic lineage attributes—particularly, dispersal ability and its interaction with the landscape over evolutionary time. Barriers therefore usually act as semi‐permeable filters, with their influence on diversification emerging from the dynamic interplay between environmental structure and lineage‐specific dispersal capacities (Waters et al., 2020).

Barriers are often treated as patterned and recurrent causal agents of allopatry, yet dispersal can also generate predictable, even synchronous events across independent lineages. The preference for barriers rests on the assumption that, unlike dispersal, they generate consistent patterns; however, the notion that dispersal events are entirely stochastic is misleading. A growing body of work shows that dispersal can display spatiotemporal regularities structured by geological history, ecological dynamics, and climatic cycles (de Queiroz, 2005; Waters et al., 2020; Fraser et al., 2022). In fact, dispersal can act as a positive generator of large‐scale biogeographic patterns, as illustrated by the concept of reticulate biogeography, where biotas merge or converge through range expansion following barrier disappearance (Ronquist, 1997), and further formalized in the notion of geodispersal, the congruent expansion of multiple lineages after barrier removal (Lieberman, 2004, 2005; Sanmartín, 2010). Together, these perspectives underscore that both barriers and dispersal can produce structured, non‐random signatures in biogeographic history.

Hovenkamp (1997, 2001) was among the first to question the analogy that treats areas as characters in biogeography and, by extension, the use of cladistic methods to infer relationships among areas, entities that are not inherently hierarchical. He reframed the problem as a shift ‘from areas to barriers’ and distinguished two inferential targets: Earth history (cross‐clade evidence for shared events) and taxon history (explaining a lineage's distribution given its phylogeny). Importantly, questioning hierarchical area histories does not invalidate the use of areas as primary biogeographic homologies; it cautions against reifying area cladograms as ontological commitments (Arias et al., 2011). Comparative assessments indicate that Hovenkamp's barrier‐focused procedures avoid unrealistic hierarchical assumptions that can undermine area‐cladistic frameworks and can recover reticulate regional histories in empirical applications. At the same time, barriers' practical value depends on subsequent correspondence tests across clades and timescales; without these, barrier hypotheses risk remaining descriptive, mirroring challenges faced by area‐based analyses (Fattorini, 2008).

The implementation of the Spatial Analysis of Vicariance (SAV) in the VIP software provides a flexible framework for identifying allopatric nodes along phylogenies (Arias et al., 2011). Instead of treating distributions as fixed inputs, VIP allows the user to adjust key parameters that directly influence the detection of disjunctions. These include the spatial resolution of the analysis (grid‐cell size), the degree of overlap tolerated between sister nodes' distributions, the rules for filling gaps around observed records (filling distance), and the possibility of downweighting or even excluding widespread taxa. Additional options allow the partial or complete removal of node distributions and the weighting of these removals within an optimization criterion that maximizes the number of disjunct nodes while minimizing ad hoc assumptions. Together, these adjustments make explicit how different analytical choices affect the recognition of candidate vicariant nodes, reinforcing the need for transparency and rigour in barrier‐centred approaches (Arias et al., 2011). Once candidate nodes are identified, two further correspondences should still be evaluated: (i) whether a given allopatry represents true vicariance by meeting spatial and temporal congruence with a barrier, and (ii) whether allopatric nodes across independent clades reflect the same historical event. These essential steps of identifying correspondences are not implemented in SAV and must be carried out manually, remaining dependent on subjective, pairwise visual comparisons of ranges. These steps are crucial for establishing homologies among barriers, and in this context taxon sampling influences the placement of potential barriers (Weiler et al., 2016).

Barriers gain explanatory force and can be treated as supported biogeographic “units” only when cross‐clade and cross‐timescale correspondences reveal consistent signals; accordingly, linking allopatric distributions to a specific barrier and inferring vicariance requires comparisons across multiple clades, a criterion that Hovenkamp (1997) formalized as “Supported Vicariant Events” (not ‘Shared Vicariance Events’, as mischaracterized by Schultz and Cracraft on p. 660). This principle parallels the logic of primary and secondary biogeographic homologies for areas of endemism (Morrone, 2001) and resonates with the challenges of congruence and pseudocongruence highlighted by Donoghue and Moore (2003) as well as Upchurch and Hunn (2002), who emphasized that apparent spatial congruence may arise from distinct historical processes rather than shared causal events. Spatially congruent allopatric sister terminals or nodes may originate at different times (temporal incongruence), while temporally congruent divergences may involve only partial spatial overlap between pairs (a kind of spatial pseudoincongruence). But, according to Fattorini (2008), two traceable vicariant events may be grouped even when the areas on each side of the nodes differ, as post‐event range change can leave descendants occupying distinct extant areas. As with area‐based analyses, barrier‐based frameworks must integrate both spatial and temporal dimensions to avoid misinterpreting biogeographic patterns. From ecological and historical perspectives, barriers are not static walls but dynamic, emergent properties of landscapes, climates, and biotic interactions (Ronquist and Sanmartín, 2011; Smith et al., 2014). Dispersal events can generate allopatry either across enduring barriers, such as rivers or straits, or through colonization of distant patches without an explicit barrier (Ronquist and Sanmartín, 2011; Morrone, 2020; Wiens, 2024). Many barriers are transient, shifting or disappearing over geological and climatic cycles, so present‐day structures represent only a simplified snapshot of past processes. Focusing solely on extant, classical barriers risks underestimating the multi‐causal and temporally stratified nature of speciation in terrestrial, freshwater, and marine taxa (Donoghue and Moore, 2003; Wiens, 2024).

In my view, recent contributions advocating a barrier‐centred framework do not advance the discussion on biogeographic units: they neither overcome the methodological limitations inherited from Hovenkamp's approach nor propose substantive improvements beyond Arias et al.'s (2011) quantitative refinements. As already emphasized by Fattorini (2008), barrier‐based analyses face the same congruence problems as area‐based approaches and, without clear operational criteria, remain largely subjective and underutilized. Schultz and Cracraft (2024) also fail to engage with key questions, such as how to operationalize available methods, how to overcome implementation problems, how to integrate temporal information from dated phylogenies, and how to stipulate homology among events inferred from different clades. Without addressing these points, their proposal adds little beyond what was already critically assessed by Fattorini (2008) or empirically implemented in Arias et al.'s (2011) SAV method. Any approach that seeks to explore mechanistic explanations in biogeography should adopt a rigorous quantitative framework. To date, the only satisfactory implementation of Hovenkamp's method remains that of Arias et al. (2011), whose refinements highlight precisely the analytical rigour needed to assess spatial and temporal correspondences. By neglecting both the criticisms raised by Fattorini and the solutions offered by Arias, the recent proposal remains conceptually appealing but operationally hollow.

## Beyond areas and barriers: an integrative perspective

Neither area‐based approaches nor barrier‐centred frameworks are inherently superior, and there is no empirical basis to claim that one or the other more closely reflects the ontology of biogeographic units. In practice, historical biogeography, whether at the level of taxon biogeography (within a lineage) or area biogeography (across clades), requires the specification of a comparative unit, be it an “area” or a “barrier” (Morrone, 2009; Parenti and Ebach, 2009). The key distinction is procedural: area‐based methods reduce incongruence a priori by generalizing distributions into predefined spatial units, whereas barrier‐based approaches confront incongruence a posteriori after identifying allopatric nodes and events. This contrast mirrors the classic delineation between *a priori* and *a posteriori* approaches in cladistic biogeography (Nelson and Platnick, 1981; Van Veller et al., 2002; Morrone, 2009).

Although Schultz and Cracraft (2024, pp. 656–657) emphasize that historical biogeography should prioritize barriers as the primary drivers of allopatry, they further propose this as the foundation for developing a mechanistic framework of biogeography (pp. 658–659). Barriers are inherently dynamic, appearing, shifting or disappearing through geological and climatic cycles, as correctly depicted in Schultz and Cracraft (2024), fig. 1a,b. Moreover, barrier homology requires strict correspondence between barrier formation and lineage divergence, as single allopatric splits are insufficient to establish vicariance. An exclusive focus on barrier detection therefore tends to relocate, rather than resolve, the ontological and methodological difficulties of establishing historical biogeographical homologies, especially when causal inference demands patterns across clades and time (Fattorini, 2008). Framing barriers as the cornerstone of a mechanistic vision of historical biogeography is problematic, since mechanistic explanations require causal processes that can be tested and generalized, yet barriers are often transient and unstable entities. Focusing exclusively on their identification may capture part of the picture, but inevitably leaves out other fundamental drivers of diversification, such as dispersal, ecological specialization and shifting connectivity.

A mechanistic biogeography should be explicitly plural and, above all, process‐agnostic, avoiding the reification of any single causal class (i.e. barriers/vicariance). As Lorcery et al. (2025) demonstrate, mechanistic eco‐evolutionary models—such as Gen3sis—integrate paleoclimate, palaeogeography, and biological processes (e.g. dispersal, speciation, and extinction) into a single framework. These models successfully recover large‐scale patterns, including the latitudinal diversity gradient and the tropics' dual ‘cradle/museum’ role. Across broad parameter combinations, they show that first‐order biogeographic signals are driven by the coupled history of climate and tectonics, rather than by any single, isolated mechanism. This can be interpreted as indicating that, even in synthetic simulated systems that explicitly incorporate multiple types of biogeographical processes, the results suggest that no single process operates in isolation or can be regarded as uniquely responsible for present‐day distributional patterns. Models should not be designed to confirm a particular theory, but to explore competing and complementary mechanisms, thereby providing the proper basis for testing spatiotemporal correspondences among processes (e.g. dispersal, vicariance, ecological filters) rather than presupposing barriers as privileged explanatory units or process. Consistently, bottom‐up biogeographical simulations in which geographic ranges are governed by only four control parameters (dispersal distance, evolutionary rate, time to speciation, competition intensity) reproduce macro‐patterns, including cradle/museum dynamics and the latitudinal diversity gradient, without a priori target fitting (Rangel et al., 2018). Envisioning mechanistic historical biogeography as driven by a single process is therefore an unwarranted simplification. Historical biogeography prioritizes lineage‐explicit reconstructions of range evolution along phylogenies and the diagnosis of particular events (dispersal, vicariance, extinction) in specific clades, whereas macroecology aims to explain aggregate patterns (e.g. richness gradients, diversification dynamics) and the processes that generate them. The demarcation is pragmatic rather than ontological; where they interrogate the same spatiotemporal histories, their process‐based inferences should be mutually consistent—persistent discordance would indicate problems of scale, data or model specification, not competing pasts.

A recurring hazard in historical biogeography is the model‐first shortcut: imposing a simplified spatial scheme and then fitting the evidence to it. Approaches that treat ‘areas’ as characters, score their fit on alternative trees, and use these scores (e.g. consistency indices across morphological and molecular phylogenies) to adjudicate among phylogenies exemplify this reductionism (see Oyston et al., 2022). Such procedures invert the logic of inference: rather than allowing phylogenetic history to inform biogeographic hypotheses, they compel phylogenetic evidence to conform to pre‐specified spatial models. The result is not only a muted historical signal but also a risk of self‐confirmation, with the model dictating the explanation instead of being tested by the data. As Ree and Sanmartín (2009) emphasized, models are heuristics for inference, not ontological commitments; when they are treated as the *explananda*, biogeography shifts from discovery to confirmation. Philosophically, this is a category mistake, confusing an operational device with a causal account, and empirically suppresses the heterogeneity of processes, time scales, and spatial contexts that biogeographic data routinely display.

Parametric approaches were a major step forward because they made the assumptions explicit and testable. DEC, BayArea, DIVALIKE, and their +J variants estimate ancestral ranges under process‐based Markov models and permit likelihood/Bayesian comparison (Landis et al., 2013; Matzke, 2014; Ree and Sanmartín, 2018). Yet, these frameworks still rest on strong idealizations, what events are allowed, how they may be combined, whether rates are homogeneous through time and among lineages, and known sensitivities persist (e.g. the ‘null range’ problem in DEC and its influence on model selection; see discussion in Ree and Sanmartín, 2018). Statistical preference among models cannot be conflated with mechanistic truth: good relative fit does not guarantee that the generating processes are correctly specified, that parameters are identifiable, or that the same model applies across clades or epochs (Ree and Sanmartín, 2009; Landis et al., 2013). Uncertainty in the tree itself further propagates into range reconstructions and model rankings (Magalhaes et al., 2021). In short, even the most sophisticated pipelines remain approximations of a messy reality, and their outputs should be treated as hypotheses whose credibility rises when they align with independent geological, paleoclimatic, and ecological evidence, not as proofs delivered by a single favoured model. A mechanistic program in historical biogeography must reckon with extinction, whose under‐modelling and poor identifiability distort inference. As Sanmartín and Meseguer (2016) emphasize, estimating extinction from extant‐only timetrees is notoriously fragile; spatially asymmetric extinction can erase ancestral ranges or masquerade as single dispersal events, while commonly used parametric frameworks (e.g. DEC) tend to under‐estimate extinction (and often dispersal), compounding these biases. The remedy is to make extinction explicit by integrating fossil evidence and environmental covariates, relaxing stationarity via time‐heterogeneous models, and testing for area‐dependent extinction across clades.

If the central problem with area‐based methods is their reliance on predefined spatial units, it is important to recognize that several approaches have been developed beyond both area‐ and barrier‐based frameworks, which do not presuppose discrete areas but instead model spatial evolution directly along lineages. Early contributions include maximum likelihood and Bayesian frameworks for continuous phylogeography, which estimate ancestral locations under diffusion processes in continuous space (Lemmon and Lemmon, 2008; Lemey et al., 2010). Bouckaert et al. (2012) applied this approach to Indo‐European languages, reconstructing origins and routes of expansion through Bayesian continuous diffusion models. Later, Nylinder et al. (2014) and Meseguer et al. (2015) extended these models by integrating dated phylogenies and dynamic dispersal scenarios, enabling more realistic spatiotemporal hypothesis testing. Quintero et al. (2015) employed similar diffusion‐based methods to investigate historical trajectories, a perspective recently expanded by Culshaw et al. (2024), who introduced a process‐based, forward‐time simulation framework to integrate more explicitly phylogenetic information into evolutionary macroecology. Building on this trajectory, Arias (2024) proposed an explicit paleogeographic framework that incorporates plate tectonics and landscape changes, while Duarte et al. (2025) introduced a site‐based method (i.e. SBEARS) that enables ancestral range estimation directly on high‐resolution grids. Despite their distinct applications, these methods converge in their effort to bypass the subjectivity of predefined area units, while still facing challenges such as model complexity, reliance on strong statistical assumptions, and limited spatial or temporal resolution.

The processes shaping biodiversity patterns are best understood as complex and emergent, marked by nonlinearity, feedbacks and spatiotemporal scale dependence, and thus not reducible to single causes (see Holland, 1992; Levin, 1998; Lorcery et al., 2025). Patterns of endemism and diversification arise from interactions among multiple drivers across space and time—often yielding equifinal outcomes that preclude single‐cause explanations (Nihei, 2008; Daru et al., 2020; Morrone, 2020). Integrative, process‐based models further show that different combinations of speciation, extinction, dispersal and ecological limits can reproduce similar macro‐patterns; treating heuristic models as ontological truths or assuming a preponderant cause therefore obscures the heterogeneity and contingency of biogeographic systems (Levin, 1998; Rangel et al., 2018; Etienne et al., 2019; Pontarp et al., 2019). Rather than imposing a single mechanistic template, progress rests on pluralist, integrative strategies that combine alternative units (areas, barriers, cenocrons, biotas sensu Morrone, 2009, 2020) and cross‐validate process inferences against replicated spatiotemporal patterns, dated phylogenies and Earth history evidence (Ree and Sanmartín, 2009). This requires making assumptions explicit, acknowledging phylogenetic and parametric uncertainty, and recognizing that emergent patterns, whether driven by dispersal, vicariance, or ecological shifts, resist reduction to fixed causal models (Ree and Sanmartín, 2009, 2018; Landis et al., 2013; Matzke, 2014; Morrone, 2020; Magalhaes et al., 2021).

Just as in M. C. Escher's woodcut ‘Another World II’ invites the viewer into a scene where multiple orientations coexist yet never resolve into a single, coherent perspective, the debate between area‐ and barrier‐based approaches in historical biogeography exposes the impossibility of capturing the full complexity of biotic history through any single analytical lens. Each framework illuminates a different facet of a larger reality; areas summarize recurring patterns of shared distribution, while barriers foreground the causal processes that restrict gene flow, yet neither can by itself encompass the multifaceted and emergent nature of diversification. Attempts to impose a purely mechanistic or reductionist model risk flattening this complexity into a partial and distorted view, obscuring the interplay of spatial, temporal, ecological, and evolutionary dimensions. Insights from the philosophy of science in practice further emphasize that causal reasoning in ecology and evolution is inherently plural and context‐dependent: what counts as a mechanism, a relevant component, or an operative interaction varies with investigative aims, scales and empirical constraints rather than obeying a fixed mechanistic template (Poliseli, 2025). Historical biogeography, therefore, should be understood not as a search for a single ‘true’ unit or universal model, but as a discipline defined by emergent patterns, where different approaches reveal complementary perspectives on a reality that, like Escher's impossible architecture, can never be fully resolved from only one vantage point.

## Conflict of interest

The author declares no conflict of interest.

## Data Availability

Data sharing not applicable to this article as no datasets were generated or analysed during the current study.
